# Ethacrynic acid improves the antitumor effects of irreversible epidermal growth factor receptor tyrosine kinase inhibitors in breast cancer

**DOI:** 10.18632/oncotarget.10846

**Published:** 2016-07-26

**Authors:** Bing Liu, XinPing Huang, YunLong Hu, TingTing Chen, BoYa Peng, NingNing Gao, ZhenChao Jin, TieLiu Jia, Na Zhang, ZhuLin Wang, GuangYi Jin

**Affiliations:** ^1^ National-Regional Key Technology Engineering Laboratory for Synthetic Biology of Medicine, Cancer Research Center, Shenzhen University, Shenzhen, China; ^2^ Department of Pharmacy, School of Medicine, Health Science Center, Shenzhen University, Shenzhen, China; ^3^ The Cancer Research Center, Shenzhen University, Shenzhen, China; ^4^ Department of Pathophysiology, Chongqing Medical University, Chongqing, China; ^5^ Shenzhen Conjugenix Pharma-Tech Co. Ltd, Guangdong, China

**Keywords:** breast cancer, EGFR, tyrosine kinases, ethacrynic acid

## Abstract

Prolonged treatment of breast cancer with epidermal growth factor receptor (EGFR) tyrosine kinase inhibitors (TKIs) often results in acquired resistance and a narrow therapeutic index. One strategy to improve the therapeutic effects of EGFR TKIs is to combine them with drugs used for other clinical indications. Ethacrynic acid (EA) is an FDA approved drug that may have antitumor effects and may enhance the cytotoxicity of chemotherapeutic agents by binding to glutathione and inhibiting WNT signaling. While the α,β-unsaturated-keto structure of EA is similar to that of irreversible TKIs, the mechanism of action of EA when combined with irreversible EGFR TKIs in breast cancer remains unknown. We therefore investigated the combination of irreversible EGFR TKIs and EA. We found that irreversible EGFR TKIs and EA synergistically inhibit breast cancer both *in vitro* and *in vivo*. The combination of EGFR TKIs and EA induces necrosis and cell cycle arrest and represses WNT/β-catenin signaling as well as MAPK-ERK1/2 signaling. We conclude that EA synergistically enhances the antitumor effects of irreversible EGFR TKIs in breast cancer.

## INTRODUCTION

Breast cancer is the most common cancer and the leading cause of cancer death among women worldwide, with about 1.7 million cases and 521,900 deaths in 2012. Although the incidence of breast cancer is much higher in the developed world, mortality rates are higher in the developing world, possibly because of the lack of affordable treatment and reduced access to mammography screening and tumor-receptor testing. Yet, even in the United States, breast cancer has a 5-year survival rate of only 23% for patients diagnosed with distant metastasis [1], [2]. Therefore, it is necessary to continue to improve the therapeutic efficiency of affordable medicine in breast cancer.

The family of HER (ERBB) receptor tyrosine kinases consists of four members: epidermal growth factor receptor (EGFR, also known as HER1 or ERBB1), HER2 (ERBB2), HER3 (ERBB3), and HER4 (ERBB4) [3]. Overexpression, mutation, or aberrant activity of these receptors contributes to the progression of breast cancer by activating intracellular signaling cascades, including the MAPK-ERK and PI3K/AKT pathways [3], [4]. *HER2* is overexpressed in 15-20% of all breast cancers and is correlated with poor prognosis. In addition, approximately 50% of all triple-negative breast cancer (TNBC) and inflammatory breast cancer overexpress *EGFR* [5]. Thus, treatments specific to different morphological types of breast cancer and relevant targets of the EGFR family are emerging as promising options.

Afatinib (BIBW-2992) and neratinib (HKI-272) are second generation irreversible EGFR family tyrosine kinase inhibitors (TKIs) that are able to covalently alkylate a specific cysteine residue close to the ATP-binding site of the receptor [6]. Unlike first-generation EGFR TKIs, such as gefitinib, clinical trials have suggested that these new drugs can overcome resistance. A recent preclinical study performed at the University of Washington identified 13 *HER2* somatic mutations in breast cancers lacking amplification of the *HER2* gene. These mutations produced a neomorphic phenotype with increased phosphorylation of EGFR or HER2 and lapatinib resistance; however, all mutant cells were sensitive to the irreversible TKI, neratinib [5], [7]. In a Phase II trial, the pan-HER inhibitor, afatinib, showed promising activity in patients with HER2+ breast cancer whose disease had progressed after trastuzumab treatment. Afatinib was also found to have anti-proliferative effects on TNBC cell lines.

The rationale for assessing afatinib in our study was based on the high EGFR expression in TNBC and the assumption that uncontrolled ERBB signaling is related to an increased oncogenic potential in TNBC subtypes. However, the results from LUX-Lung 2 and 3 trials, with a median progression free survival (PFS) of 12–14 months with first-line afatinib treatment in EGFR-mutant non-small cell lung cancer, demonstrated that acquired resistance (AR) is still a major clinical issue in treatment with afatinib, due to crosstalk between pathways. These findings suggest that afatinib and neratinib administered at current clinically recommended doses may not be sufficient to effectively suppress some cancers. Hence, it is absolutely essential to find new strategies to improve the therapeutic effects of these drugs and overcome AR.

Recently, it was reported that ethacrynic acid (EA), which is used clinically as a diuretic agent, inhibits glutathione S-transferase P1-1 (GSTP1-1) and WNT activity [8], [9], [10]. Glutathione-S-transferase (GST) is overexpressed in human tumors in the reduced form glutathione (GSH) and binds to electrophilic compounds, leading to detoxification of the cells. As a result, the binding of EA to GSH enhances the cytotoxicity of chemotherapeutic agents [9]. Additionally, aberrant activation of the WNT signaling pathway has been detected in breast tumors, and the expression of Frizzled-related protein 1 (sFRP1), a secreted factor that inhibits WNT signaling, is downregulated in many breast tumors and associated with poor prognosis [11]. Interestingly, the chemical structure of α,β-unsaturated keto functional group of EA is similar to that of irreversible TKIs, as shown below; however, the role of EA's combinational function on the irreversible EGFR TKIs in breast cancer remains unknown. Thus, we asked whether EA could potentiate the antitumor effects of irreversible EGFR TKIs in breast cancer.

**Figure:**
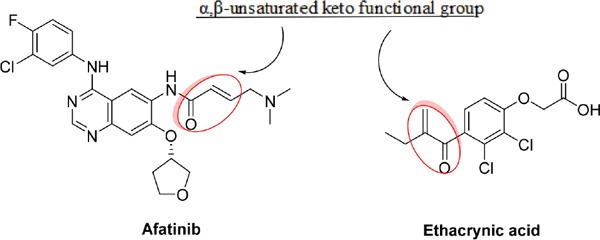


## RESULTS

### The cytotoxic effect of irreversible EGFR TKIs and ethacrynic acid on breast cancer cell lines

**Figure 1:**
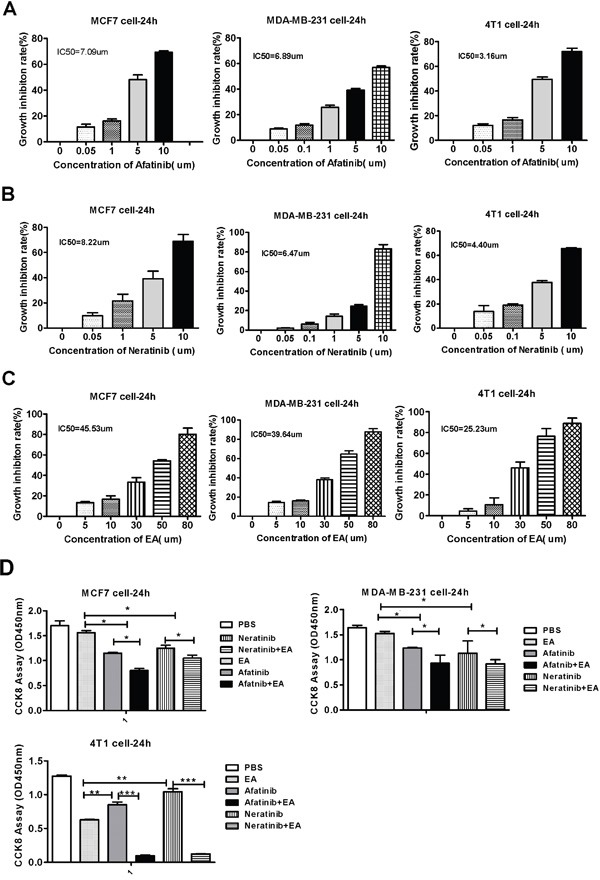
Cytoxicity of irreversible EGFR TKIS and ethacrynic acid on breast cells lines **A.** Mean IC_50_ value of Afatinib. **B.** Mean IC_50_ value of Neritinib. **C.** Mean IC_50_ value of Ethacrvnic Acid (EA). **D.** The effect ofcombination EA and afatinib on 4T1, MDA-MB-231, MCF-7 tumor cell lines. *IC50 is the mean concentration of drug that reduced cell survival by 50% in at least two experiments. Data are shown as mean ± SD (n=6) of one representative experiment. Similar results were obtained in three experiments. *p < 0.05; **p < 0.01;*** p < 0.001.

To investigate the toxicity of irreversible EGFR TKIS (afatinib and neratinib) and ethacrynic acid (EA) on breast cells lines, MCF7, MDA-MB-321 and 4T1 cells were treated with afatinib, neratinib and EA at different concentrations for 24h. Cytotoxicity was calculated based on cell viability as determined by CCK8 assays. As shown in Figure 1A-1C, the rate of cell death increased with drug concentration in all three cell lines. The half maximal inhibitory concentrations (IC50) of afatinib, neratinb and EA for MCF7, MDA-MB-231 and 4T1 in 24h were tested by *CCK8* assay. We chose the 30%~40% inhibitory concentration of afatinib (4 μm), neratinib (4 μm) and EA (25 μm) in these cells for subsequent experiments.

### Combination treatment of irreversible EGFR TKIs with ethacrynic acid has synergistic antitumor effects on breast cancer cells

We next determined the effect of combination treatment with irreversible EGFR TKIs and EA on inhibiting the proliferation of breast cancer cells using CCK8 assays. As shown in Figure 1D, while treatment with afatinib or neratinib alone modestly reduced the viability of MCF7, MDA-MB-231 and 4T1 cells, combining these drugs with EA resulted in decreased cell viability (*P*<0.05). Among the three cell types, the combination effect on 4T1 cells was the most dramatic (*P*<0.001). These combination effects were further evaluated on the basis of the coefficient of drug interaction (CDI). As shown in Table 1, the CDI of afatinib or neratinib in combination with EA in the three breast cancer cells was less than 1, which means the drugs acted synergistically. The synergistic effect on 4T1 cell was the most obvious (CDI_AE_=0.23, CDI_NE_=0.24). In addition, the effect of afatinib combined with EA was more pronounced than the effect of neratinib combined with EA.

**Table 1:** The coefficient of drug interaction (CDI)

Cell	EA	Afatinib	A+E	CDI_AE_	Neratinib	N+E	CDI_NE_
**MCF7**	0.92	0.67	0.47	0.76	0.73	0.58	0.87
**MDA-MB-231**	0.93	0.75	0.73	0.71	0.71	0.56	0.85
**4T1**	0.49	0.66	0.07	0.23	0.82	0.09	0.24

Combination treatment with irreversible EGFR TKIs and ethacrynic acid (EA) had a synergistic antitumor effect in breast cancer cells. The results are from one representative experiment. Similar results were obtained in three experiments.

### Combination treatment with irreversible EGFR TKIs and ethacrynic acid inhibits breast cancer cell cycle progression and induce cell death to repress tumor growth

**Figure 2:**
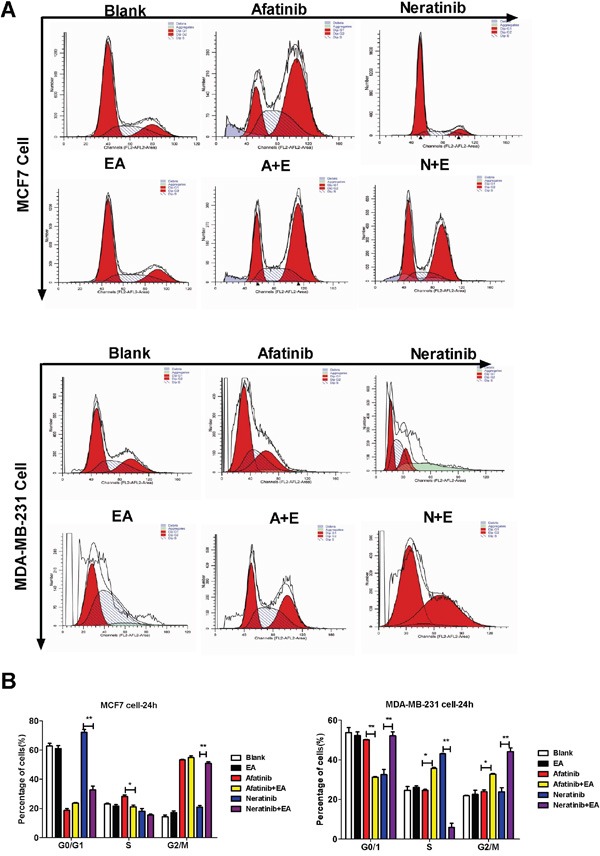
Combination of TKIs with EA reduced tumor cell proliferation by inhibiting cell cycle progression **A.** 4T1, MDA-MB-231, MCF-7 cells were treated with afatinib/neratinib and/or EA for 24 hours after the cells were harvested and analyzed by FACS. **B.** The calculated cell cycle distribution. Data are shown as mean ± SD (n=6) of one representative experiment. Similar results were obtained in three experiments. *p < 0.05; **p < 0.01;*** p < 0.001.

**Figure 3:**
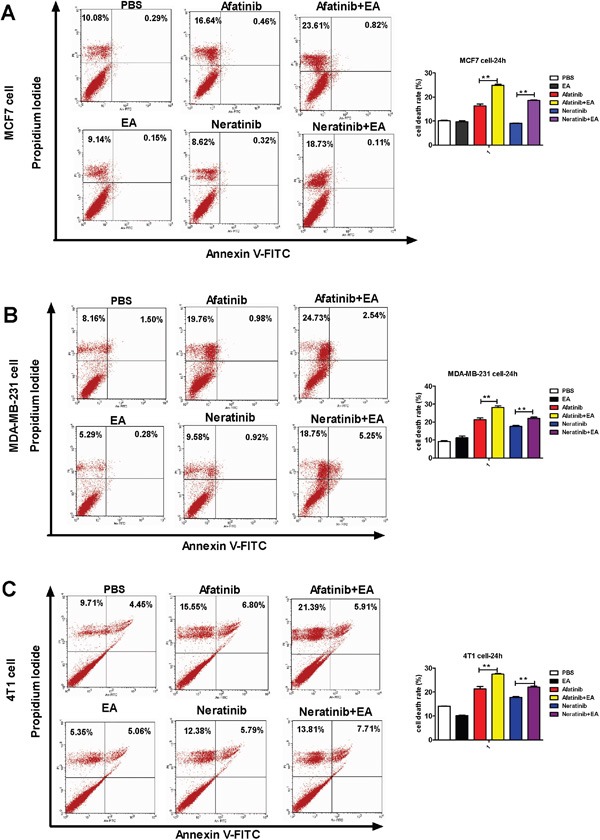
Combination of TKIs with EA induced tumor cell apoptosis The tumor cells were stimulated with 4 μg afatinib/neratinib and/or 30 μg EA for 24 hours, stained with annexin V and propidium iodide, and measured by FACS. Data are shown as mean ± SD (n=6) of one representative experiment. Similar results were obtained in three experiments. *p < 0.05; **p < 0.01;*** p < 0.001.

To analyze the mechanism of synergistic inhibitory effects induced by the combination of either afatinib or neratinib with EA, we examined whether these changes of tumor cell growth were related to the cell cycle and apoptosis. Cell cycle analysis on the breast cancer cell lines indicated that afatinib combined with EA blocked cell cycle progression of MCF7 cells at G2/M phase and MDA-MB-231 cells at S phase and G2/M phase. Neratinib combined with EA blocked cell cycle progression of both MCF7 and MDA-MB-231 cells at G2/M phase (P<0.01; Figure 2A-2B). In addition, as shown in Figure 3A-3C, when compared with afatinib, neratinib, alone, the number of necrotic cells was increased in all breast cancer cell lines with the combination of afatinib or neratinib with EA (P < 0.01). These results indicate that the combination groups inhibit cancer cells growth by blocking cell cycle progression and inducing necrotic.

### Combination treatment with irreversible EGFR TKIs and ethacrynic acid enhances antitumor effects *in vivo*

**Figure 4:**
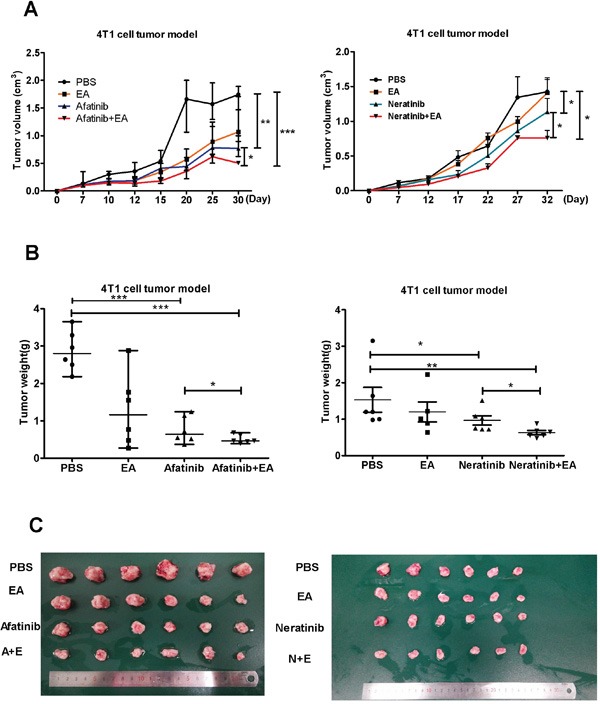
Combination of TKIs with EA suppressed tumor growth *in vivo* **A.** The change in tumor volume (mean±SD) of mice from 3 independent experiments. **B.** Tumor weights after quintic treatment. **C.** Image showing method of tumor removal in one representative experiment. Data are shown as mean±SD (n=6) of one representative experiment. Similar results were obtained in three experiments. *p < 0.05; **p < 0.01;*** p < 0.001.

To evaluate whether combined treatment with EA and either afatinib or neratinib had stronger anti-tumor effects *in vivo*, 4T1 breast cancer cells were implanted subcutaneously into the back of syngeneic Balb/c mice. When all these tumors reached 62.5mm^3^ in volume at least, mice were treated with afatinib (20 mg/kg) or neratinib (20 mg/kg) alone or together with EA (250 ug/day) for 3 weeks [16], [17], [18]. Consistent with our *in vitro* results, monotherapy with afatinib or neratinib decreased tumor growth, but treatment with the combination of afatinib or neratinib and EA led to marked tumor shrinkage. It is notable that afatinib combined with EA improved the tumor growth inhibition rate of afatinib alone by 34.2%. While neratinib combined with EA improved the inhibition of tumor growth compared to neratinib alone by only 22.4% (Figure 4).

### Combination treatment with irreversible EGFR TKIs and ethacrynic acid co-suppresses the WNT and MAPK signaling pathways to repress tumor growth

**Figure 5:**
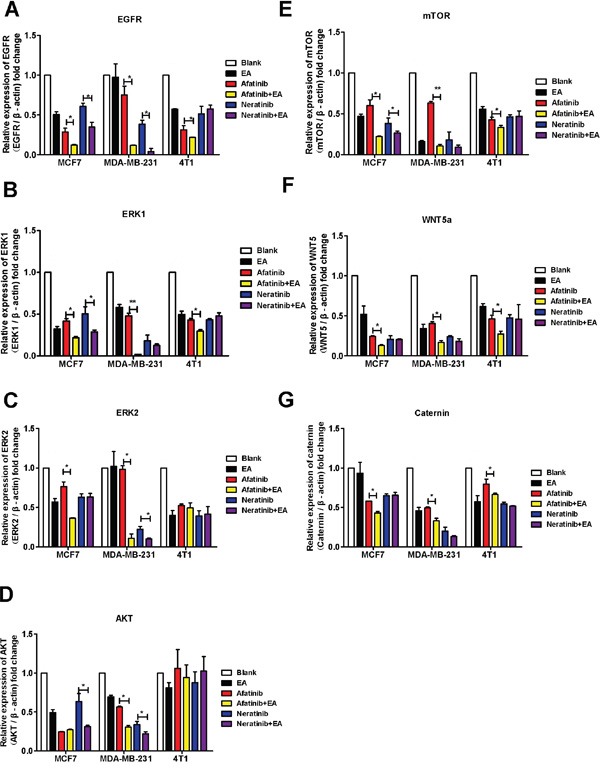
Combination of TKIs with EA inhibited the EGFR pathway MCF-7, MDA-MB-231 and 4T1 cells were analyzed by real-time pRT-PCR after stimulation with 4μg afatinib/neratinib and/or 30μg EA for 24 hours. **A-D.** show the impact on EGFR, ERK1, ERK2, AKT, mTOR, WNT5A, and β-catenin. Data are shown as mean ± SD (n=6) of one representative experiment. The images are representative of one experiment. Similar results were obtained in three experiments. *p < 0.05; **p < 0.01;*** p < 0.001.

**Figure 6:**
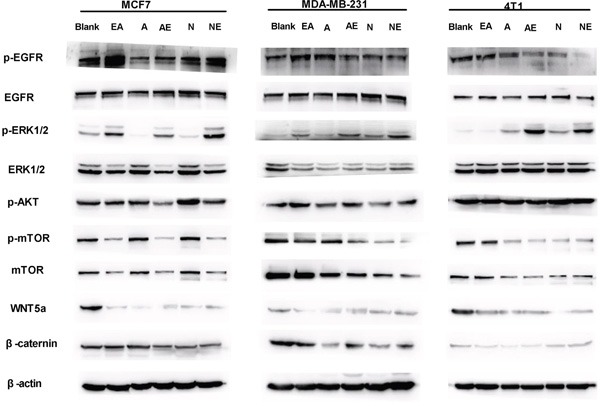
Effect on expression of proteins in the WNT/β-catenin and MAPK-ERK1/2-mTOR pathways Lysates from indicated breast cancer cell lines were analyzed by SDS-PAGE followed with specific antibodies to EGFR, p-EGFR, ERK1/2, p-ERK2, mTOR, p-mTOR, p-AKT, WNT5A, β-actin, and β-catenin. Data are shown as mean ± SD (n=6) of one representative experiment. The images are representative of one experiment. Similar results were obtained in three experiments. *p < 0.05; **p < 0.01;*** p < 0.001.

Autocrine WNT signaling contributes to breast cancer cell proliferation via the canonical WNT pathway and EGFR transactivation [11]. Therefore, we investigated whether the molecular mechanism of the synergistic inhibitory effect induced by the combination of afatinib or neratinib with EA was dependent upon the suppression of MAPK-ERK1/2 signaling. As shown in Figure 5, qRT-PCR revealed that afatinib combined with EA suppressed the *ERK1*, *ERK2, EGFR, AKT, mTOR, WNT5a and caternin* mRNA in MCF7 and MDA-MB-231 cells compared to irreversible EGFR TKIs alone (Figure 5A-5G), but it is interesting that combined irreversible EGFR TKIs with EA didn't have abvious suppression on ERK2 in 4T1 (Figure 5C). The combination of neratinib could suppress EGFR, AKT mRNA in MCF7 and MDA-MB-231 cells compared to neratinib alone (Figure 5A&5D). Consistent with the mRNA results, as shown in Figure 6, combined with EA in MCF7 and MDA-MB-231 cells increased p-ERK1/2 expression and reduced expression of ERK1, p-mTOR, mTOR, and WNT5a when compared with afatinib or neratinib alone, while levels of p-mTOR, and mTOR in 4T1 cells also showed similar trends. Interestingly, although EA or it combined with TKIs decreased the expression of ERK1/2 in human breast cancer cell lines(MCF7;MDA-MB-231) but the p-ERK1/2 was increased.

## DISCUSSION

Cancer treatments based on blocking EGFR signaling are emerging as promising alternatives; however, tyrosine kinase inhibitors (TKIs) show only modest effects on most cancer types. Particularly for patients with advanced tumors, single-agent TKI therapy has a narrow therapeutic index. In addition, the majority of positive responses to TKIs is merely palliative and often unpredictable. Unfortunately, a large number of patients fail to achieve initial responses or improvements in survival with currently available TKIs [19]. Thus, it is urgent to find a new strategy to improve therapeutic effects on cancer patients. In this study, we investigated the effects of ethacrynic acid (EA) combined with irreversible EGFR TKIs on breast cancer both *in vitro* and *in vivo*. We found that a safe dose of EA extends the therapeutic effect of irreversible EGFR TKIs on breast cancer.

Previous studies have demonstrated that EA and its analogues are glutathione transferase P1-1 (GSTP1-1) and WNT inhibitors and that EA alone induces cell death in malignant cells at high concentrations. EA exhibits selective toxicity to chronic lymphocytic leukemia cells by inhibiting the Wnt/β-catenin signaling pathway [8]. EA analogues inhibit the migration of human prostate cancer and breast cancer cell lines, which provide a good model system to study migration and invasion since they represent metastatic cancer [20], [21]. Zhang *et al*. (2013) [22] also reported on a novel oxadiazole analogue of EA, EA6r, that inhibits the proliferation of a range of human colon, leukemia, prostate, lung, breast, ovarian, and cervical tumor cell lines by inducing cell apoptosis and S-phase arrest. The antitumor activity of this EA analogue was also shown in tumor xenografts. These results are consistent with our q-PCR data showing that EA inhibited the expression of genes downstream of WNT5a/β-catenin and MAPK-ERK1/2-mTOR.

GSTP1-1 is overexpressed in tumor cells, suggesting that it may contribute to their drug resistance. Based on that, inhibitors of GSTP1-1 are expected to counteract drug resistance and serve as adjuvants in cancer chemotherapy by increasing efficacy. Finding other useful inhibitors among compounds used for other clinical indications might be a shortcut to clinical applications [23]. For example, EA improves the antitumor effects of arsenic trioxide (ATO) in myeloid leukemia and lymphoma cells by inducing apoptosis and inhibiting GSTP1-1 [24]. In addition, EA combined with selenite prevents the development of GST-dependent cisplatin resistance in a model of human H562 small cell lung cancer [25]. Analogous to these previous studies, our results showed a synergistic effect of EA with irreversible EGFR TKIs in breast cancer. The combination treatment not only inhibited proliferation *in vitro* but also repressed tumor growth *in vivo*. Specifically, afatinib combined with EA remarkably reduced tumor volume 30% more than treatment with afatinib or neratinib alone.

TKIs primarily activate MAPK-ERK and PI3K/AKT/mTOR pathways, while EA acts on the WNT/β-catenin pathway. One study has investigated the role of WNT/β-catenin in breast cancer and found that WNT pathway signaling was increased in cancer tissue compared to normal breast. Furthermore, expression of WNT pathway genes correlated with estrogen receptor (ER) expression, especially WNT5a and WNT5b, with approximately 50% of breast cancer tissues overexpressing these genes [26]. Additionally, there was a finding that autocrine WNT signaling activates the extracellular signal-regulated kinase 1/2 (ERK1/2) pathway in mammary epithelial cells via EGFR transactivation [11]. As shown in Figure 5 & 6, the mRNA and protein expression of ERK1 and ERK2 in MCF7 cells were inhibited by combination EA treatment more than with afatinib or neratinib alone. This is likely because MCF7 cells expressing ER are more sensitive to EA alone compared to other agents. Moreover, expression of mTOR, which is downstream of ERK1/2, was lower in MCF7 cells treated with EA than in cells treated with afatinib or neratinib alone. The inhibitory effects of EA in TNBC cells were weaker than in MCF7 cells, probably because 50% of TNBC cells overexpress EGFR, and afatinib and neratinib also target EGFR. It is clear from our results that the combination of afatinib or neratinib with EA also improves antitumor action by enhancing the inhibitory effects of the ERK or mTOR pathways. In our study, pERK was also increased by combination treatment. Anti-tumor drugs also activate p38 and pERK1/2 in the MAPK signaling pathway, and this activity may be related to autophagy [10]. Therefore the mechanism of pERK1/2 in cancer therapy needs further study.

In summary, we demonstrate for the first time that EA synergistically augments the antitumor effects of irreversible EGFR TKIs in breast cancer. These effects were seen not only in HER2 or ER positive breast cancers, but also in TNBC cells. Further investigation is required to fully reveal the effects of these combination treatments on different breast cancer subtypes. The combination therapeutic effects of EA with irreversible EGFR TKIs in breast cancer are worthy of clinical evaluation in humans.

## MATERIALS AND METHODS

### Cell culture and reagents

All cells were obtained from the Institute of Biochemistry and Cell Biology of Chinese Academy of Science (Shanghai, China). Human breast cancer cell lines (MCF7 and MDA-MB-231) and mouse breast cancer cells (4T1) were maintained in RPMI 1640 with 10% fetal bovine serum (FBS) (PAA Laboratories, Pasching, Australia) in a humidified atmosphere containing 5% CO_2_ at 37°C. Afatinib and neratinib were purchased from Selleck Chemicals company (USA), and ethacrynic acid was obtained from Sigma (USA).

### Cell proliferation assay

The *in vitro* cell proliferation of breast cancer cell lines were determined by WST-8 staining with the Cell Counting Kit-8 (Dojinodo, Shanghai, China) according to the manufacturer's instructions. Briefly, cells were seeded in 96-well plates in a density of 6000 cells/well and incubated for 24 h followed treated with afatinib, neratinib with or EA for 24h. Then cells were stained with WST-8 followed by determination of OD _450 nm_ with a reference wavelength at 600 nm. The data were analyzed using Graphpad Prism 5 software to obtain the IC50 [12].

### Cell cycle staging

Cells were fixed in 70% ethanol at 4°C for 24h, stained with 50 ug/ml propidium iodide and 1 mg/ml ribonuclease A (Kaiji, Nanjing, China) at room temperature for 30min, and analyzed by FACS Caliber (BD Bioscience, MA, USA).

### Apoptosis assay

24h after treatment with drugs, cells were harvested, washed, re-suspended in the binding buffer, and examined with the Vybrant Apoptosis Assay kit (Invitrogen, Carlsbad, CA, USA). Stained cells were detected by FACS (FACScalibur, Becton Dickinson, Mountain View, CA, USA) as previously described [13]. Annexin V-positive cells were regarded as apoptotic.

### *In vivo* studies

For mouse studies, female BALB/c mice (5-6 weeks of age) were obtained from Guangdong province Laboratory Animal Center (Guangzhou, China). 4T1 cells (2×10^5^) were suspended in 0.1ml phosphate buffered saline (PBS) and then injected subcutaneously into the backs of the mice. Tumor growth was measured over the course of 30 days, and tumor volume was calculated according to the formula: volume = 0.5× length × width^2^. All experiments were performed in accordance with the Guide for the Care and Use of Laboratory Animals, with the approval of the Shenzhen University, Shenzhen, China.

### Coefficient of drug interaction (CDI)

The coefficient of drug interaction (CDI) was used to analyze the synergistically inhibitory effect of drug combinations. CDI was calculated as follows: CDI = AB/(A × B). According to the absorbance of each group, AB is the ratio of the combination groups to the control group; A or B is the ratio of a single agent group to control groups. Thus a CDI value less than, equal to, or greater than 1 indicates that the drugs are synergistic, additive, or antagonistic, respectively. A CDI less than 0.7 indicates that the drugs are significantly synergistic [14].

### RNA extraction and quantitative RT-PCR

Total RNA, including miRNA, was extracted using TRIzol reagent (Invitrogen, Carlsbad, CA, USA) according to the manufacturer's instructions. cDNAs were synthesized using ReverTra Ace qPCR RT kit (FSQ-101; Toyobo, Kagoshima, Japan). Real-time PCR analyses were performed with Thunderbird SYBR qPCR mix (QPS-201; Toyobo) on an MxPro Mx3000P Sequence Detection system (Stratagene, La Jolla, CA, USA). β-actin was used as an internal normalized reference, and fold changes were calculated by relative quantification (2^−ΔΔCt^). The primer sequences are shown in Table 2.

**Table 2:** Primer sequences

Primer ID	Sequence(5′to 3′)
Human-EGFR-F	AGGCACGAGTAACAAGCTCAC
Human-EGFR-R	ATGAGGACATAACCAGCCACC
mouse-EGFR-F	ATGAAAACACCTATGCCTTAGCC
mouse-EGFR-R	TAAGTTCCGCATGGGCAGTTC
human-mTOR-F	ATGCTTGGAACCGGACCTG
human-mTOR-R	TCTTGACTCATCTCTCGGAGTT
mouse-mTOR-F	CAGTTCGCCAGTGGACTGAAG
mouse-mTOR-R	GCTGGTCATAGAAGCGAGTAGAC
human-WNT5-F	ATTCTTGGTGGTCGCTAGGTA
human-WNT5-R	CGCCTTCTCCGATGTACTGC
mouse-WNT5a-F	CAACTGGCAGGACTTTCTCAA
mouse-WNT5a-R	CCTTCTCCAATGTACTGCATGTG
mouse-beta-actin-F	GGCTGTATTCCCCTCCATCG
mouse-beta-actin-R	CCAGTTGGTAACAATGCCATGT
human-beta-actin-F	CATGTACGTTGCTATCCAGGC
human-beta-actin-R	CTCCTTAATGTCACGCACGAT
human-beta-catenin-F	CATCTACACAGTTTGATGCTGCT
human-beta-catenin-R	GCAGTTTTGTCAGTTCAGGGA
mouse-beta-catenin-F	ATGGAGCCGGACAGAAAAGC
mouse-beta-catenin-R	TGGGAGGTGTCAACATCTTCTT
mouse-AKT1-F	ATGAACGACGTAGCCATTGTG
mouse-AKT1-R	TTGTAGCCAATAAAGGTGCCAT
human-AKT1-F	AGCGACGTGGCTATTGTGAAG
human-AKT1-R	GCCATCATTCTTGAGGAGGAAGT

* Primers used for PCR amplification

### Western blots

Antibodies for EGFR, p-EGFR, HER2, p-HER2, ERK1/2, p-ERK1/2, PI3K, p-PI3K, mTOR, p-mTOR, p-AKT, Wnt5a/b, β-catenin, β-actin, were purchased from Cell Signaling Technology (MA, USA). Cells were harvested and lysed with M-PER Protein Extraction Reagent (Pierce, IL, USA) supplemented with protease inhibitor cocktail. Protein concentrations of the extracts were measured using the BCA assay (Pierce, CA, USA) and equalized with the extraction reagent. Equal amount of the extracts were loaded and subjected to SDS-PAGE, transferred onto nitrocellulose membranes, then blotted as previously described [15].

### Statistical Analysis

Data are presented as mean ± standard deviation (S.D.) of one representative experiment. Unless otherwise noted, the differences between groups were analyzed by one-way analysis of variance (ANOVA) when there were more than two groups. In all cases, differences were considered statistically significant at *p*<0.05. All analyses were performed using SPSS17.0 software (Chicago, USA).
